# Oral Health Status Among Deaf Children in Cuttack City: A Cross-Sectional Study

**DOI:** 10.7759/cureus.72236

**Published:** 2024-10-23

**Authors:** Aseema Samal, Ipseeta Menon, Kunal Jha, Arpita Singh, Anushka Saxsena

**Affiliations:** 1 Department of Public Health Dentistry, Kalinga Institute of Dental Sciences, Kalinga Institute of Industrial Technology (KIIT) Deemed to be University, Bhubaneswar, IND

**Keywords:** children, deaf and mute, dental caries, hearing impaired, oral health

## Abstract

Aim

The aim was to assess the oral health status of hard-of-hearing children using the WHO Oral Health Assessment Form for Children 2013 and to evaluate their dietary and oral hygiene practices using the Oral Health Questionnaire given by the same.

Methodology

A cross-sectional study was conducted among 90 hard-of-hearing children aged five to 19 years in Cuttack city. The World Health Organization oral health assessment form 2013 for children was used for data collection. Type-III clinical examination were carried out using a mouth mirror, explorer and WHO periodontal probe. The resulting data were entered into Excel sheets (Microsoft, Redmond, WA, USA) and transferred to SPSS version 25 software (IBM Corp., Armonk, NY, USA). The chi-square test and multiple logistic regression analysis were used.

Results

Forty-nine children were aged between 13-16 years of age. Most of them had profound level of hearing loss (68%). The prevalence of dental caries was found to be 76.9% and 82.4% suffered from gingival bleeding. The logistic regression analysis demonstrated a significant association between the level of hearing loss and dental caries presence (p = 0.027), suggesting that children with higher levels of hearing loss may be more prone to dental caries. However, age (p = 0.882) and gender (p = 0.859) did not exhibit significant associations with dental caries presence. The age group has a significant association with gingival bleeding (p = 0.001), while gender shows a borderline significance (p = 0.090). However, the level of hearing loss does not appear to be significantly associated with gingival bleeding (p = 0.556).

Conclusion

In this study, the oral health status of these children was found to be considerably poor. There is a considerable need for treatment and prevention of oral disease among this population.

## Introduction

Disability has become one of the crucial aspects of human life. It is estimated that around 1.3 billion people (or 1 in 6 persons) experience a major disability [1]. Underprivileged children, socially excluded, differently abled, or having specific requirements bear most of the burden of oral diseases. Only 61% of the children with disabilities aged five to 19 years are attending educational institutions, out of which 20% of the disabled in the age group 0-19 years have a hearing disability [2].

Disability has proven to be a silent influence to poor oral health and other dental problems. According to WHO estimates, around 63 million persons in India suffer from Significant Auditory Impairment, with the estimated prevalence at 6.3% of the Indian population [3]. Numerous studies have shown that, compared to other members of the general population, people with disabilities have higher rates of dental illness, missing teeth or prolonged retained teeth and access to dental care [4]. Despite various initiatives by the government such as "Health for All" and "Universal Health Coverage," these vulnerable populations are more likely to suffer from poor oral health than healthy youngsters [5].

Communication disorders are broadly characterized as speech, language, and hearing disorders. Hearing impairment inhibits speech and linguistic outcomes, reduces cognitive abilities and mental growth and maturation and generally impedes scholastic progress in school [6]. The most prevalent unmet need among people with disabilities is oral health care. They tend to have poor oral health due to lack of knowledge regarding oral hygiene practices, diet, parental neglect, socioeconomic crisis and communication barriers. They are at greater risk of having dental caries, gingival problems and several oral diseases than the general population.

Oral health enhancement in a population begins with the collecting of epidemiological data, which aids in understanding community requirements, planning treatment and preventative approaches and monitoring the growth of the situation through time.

Since majority of the decisions are made by their parents or caregivers, there’s a need to educate them with specific oral health education that is appropriate to their needs and challenges [7]. Children this age are shown to have poorer oral health and a higher prevalence of gingival problems and dental caries [8]. Due to disability there is an increasing risk of developing oral diseases. Since parents tend to focus on the general health of their children, they overlook prioritizing their oral health [9].

Hence an accurate oral health assessment can improve the primary care and provide accessible oral health outcomes. The present study is conducted to assess the oral health status, knowledge, attitude, and oral hygiene practices of hard-of-hearing children of Cuttack district in Odisha.

## Materials and methods

A cross-sectional study was carried out in Cuttack City, Odisha, to assess oral health status and evaluate the oral health condition as well as the knowledge, attitude, and oral hygiene practices of hard-of-hearing children. The patients were taken up using multistage random sampling. A total of 10 schools were selected, which contained a total defined population. The schools were divided into clusters. Two clusters were chosen randomly. Population from within these randomly selected clusters formed the sample size. The sample size was set at 90, out of which 20 children were taken for the pilot study to test the feasibility and applicability of the questionnaire and clinical examination. The sample size was calculated assuming effect size (ρ)=0.36 and α error=0.05 with 95% confidence interval using G power software 3.1.9.7 version.

The study included individuals aged five to 19 years. Oral examinations and questionnaires were administered after written informed consent was obtained from the parents and teachers. Excluded were those who were uncooperative or suffering from chronic systemic diseases. Data on demographic details, dietary habits, and oral hygiene practices were collected using a validated questionnaire, Oral Health Questionnaire for Children (Appendices 1-4), with caretakers using sign language to explain the questions and the investigator recording the answers. A mouth mirror, explorer and a WHO periodontal probe were used to do the clinical evaluation. The oral health status of all children was assessed according to WHO Oral Health Assessment Form for Children (2013) (Appendix 1). Type III clinical examination was carried out as per ADA specification.

Calibration of the examiner

The primary investigator (A.S.) and a recorder (A.S.) from the public health dentistry department of the Kalinga Institute of Dental Science, Kalinga Institute of Industrial Technology (KIIT), Bhubaneswar, were suitably trained and calibrated to administer the interventions and record the codes by an expert. The group received interventions from a single calibrated primary investigator, and a single recorder coded each criteria in the appropriate recording proforma and questionnaire. The inter-rater reliability was assessed and the value was found to be 0.80 (Cohen's kappa). Participants will be made to sit on an ordinary chair and the examination will be carried out under adequate illumination.

Statistical analysis

The data was analysed using the statistical software Stata version 14 (StataCorp., College Station, TX, USA). Descriptive analysis was done using frequency distribution. to find the association between the gender, age group, and oral health parameters. Logistic regression analysis of dental caries and gingival bleeding presence. The confidence interval was set at 95% and alpha error was assumed at 0.05.

Ethical clearance

Before starting the investigation, ethical clearance was obtained by the Kalinga Institute of Medical Sciences Bhubaneswar with the reference no. KIIT/KIMS/IEC/1314/2023 and also the scientific research committee of the Kalinga Institute of Dental Sciences.

## Results

Table 1 shows the distribution of children on the basis of Age, Sex, and Level of Hearing Loss. The mean age of the children was found to be 15.45±2.51. Most of the children were male (61.5%). Minimum sample was calculated to be 90, though we have taken 91 children as the actual sample size for the study. Around 62 children out of 91 children had profound level of hearing loss (>81dB), 27 children had severe level of hearing loss and only two children had moderate level of hearing loss. The mean dental caries score was found to be 3.47±3 according to the WHO oral health assessment 2013 proforma for children. The mean gingival bleeding score was found to be 6.85±4.9. Most of the children used toothbrushes and fluoridated toothpaste to clean their teeth.

**Table 1:** Distribution of Study Participants based on Age, Sex, and Level of Hearing Loss

Characteristics	Frequency	Percentage
Age group (years)		
9-12	7	7.69
13-16	49	53.85
≥ 17	35	38.46
Sex		
Male	56	61.54
Female	35	38.46
Level of hearing loss		
Moderate (41‑60)	2	2.20
Severe (61‑80)	27	29.67
Profound (>81)	62	68.13

Table 2 represents the responses on Oral Health, Dental Practices, and Dietary Habits among study participants. Most of the children (39.56%) perceive their oral hygiene to be average and 24% of children perceive to have a poor oral hygiene, 9.89% of children perceived to have very poor oral hygiene and only 17.5% of children responded to have good oral hygiene. Around 46% children described their gums health to be average and only 14% children believed to have good healthy gums. Around 18.6% believed to have poor or bleeding gums.

**Table 2:** Responses on Oral Health, Dental Practices, and Dietary Habits Among Study Participants

Questions	Frequency	Percentage
How would you describe the health of your teeth?		
Very good	8	8.79
Good	16	17.58
Average	36	39.56
Poor	22	24.18
Very poor	9	9.89
How would you describe the health of your gums?		
Very good	12	13.19
Good	13	14.29
Average	42	46.15
Poor	17	18.68
Very poor	7	7.69
How often during past 12 month did you have tooth ache or feel discomfort due to your teeth?		
Often	2	2.20
Occasionally	20	21.98
Rarely	6	6.59
Never	61	67.03
Don’t know	2	2.20
How often you go to dentist during past 1 year?		
I had no visit to dentist during the past 12 months	8	8.79
I have never received dental care/visited a dentis	82	90.11
I don’t know/don’t remember	1	1.10
How often do you clean your teeth?		
Never	2	2.20
Several times a week (2–6 times)	1	1.10
Once a day	87	95.60
2 or more times a day	1	1.10
I am not satisfied with the appearance of my teeth?		
Yes	1	1.10
No	90	98.90
I often avoid smiling and laughing because of my teeth		
Yes	1	1.10
No	90	98.90
Other children make fun of my teeth		
Yes	1	1.10
No	90	98.90
Toothache or discomfort forced me to miss school/classes		
Yes	0	0
No	91	100
I have difficulty biting hard foods		
Yes	5	5.49
No	86	94.51
I have difficulty in chewing		
Yes	8	8.79
No	83	
Fresh Fruit intake		
Never	2	2.20
Several times a month	89	97.80
Biscuits, cakes, buns		
Several times a week	13	14.29
Once a day	58	63.74
Several times a day	20	21.98
Lemonade/soft drinks		
Several times a month	91	100
Jam/honey		
Several times a month	91	100
Chewing gum		
Never	91	100
Sweets/candy		
Once a week	5	5.49
Several times a week	53	58.24
Once a day	29	31.87
Several times a day	4	4.40
Milk+sugar		
Several times a month	4	4.40
Once a week	10	10.99
Several times a week	77	84.62
Tea+sugar		
Never	91	100
Coffee+sugar		
Never	91	100
Cigarettes		
Never	91	100
Chewing tobacco		
Never	84	92.31
Several times a week	3	3.30
Once a day	4	4.40

Table 3 shows the oral health status and intervention urgency among the study participants. Around 77% of children had dental caries and also 82.42% of children had gingival bleeding. Eleven percent of children also had missing teeth due to caries and few were missing due to congenital deformities. Out of 91 children, seven children had enamel fluorosis. Only nine children had dental trauma in their past. About 44 children needed prompt treatment such as scaling, 23 children needed immediate treatment due to pain/infection of dental origin.

**Table 3:** Oral health Status and Intervention Urgency Among Study Participants

Oral Health status	Frequency	Percentage
Dental caries		
Yes	70	76.92
No	21	23.08
Gingival bleeding		
Yes	75	82.42
No	16	17.58
Missing teeth		
Yes	10	10.99
No	81	89.01
Enamel Fluorosis		
Yes	7	7.7
No	84	92.31
Dental Trauma		
Yes	9	9.89
No	81	89.01
Excluded tooth	1	1.10
Intervention Urgency		
No treatment needed	10	10.99
Prevention or routine treatment needed	14	15.38
Prompt treatment (including scaling) needed	44	48.35
Immediate (urgent) treatment needed due to pain or infection of dental and/or oral origin	23	25.27

Table 4 demonstrates the distribution of children with presence of gingival bleeding according to their age, gender and level of hearing loss. A statistical significant difference is seen between age and presence of gingival bleeding. There was no statistical significant difference seen between gender and bleeding gums of children, neither any association between gingival bleeding and hearing loss.

**Table 4:** Presence of Gingival Bleeding Among the Study Participants by Age group, Gender and Level of Hearing Loss

	Yes n (%)	No n (%)	χ2	P value
Age group			11.2320	0.004
9-12	7 (100)	0 (0)		
13-16	45 (91.84)	4 (8.16)		
≥ 17	23 (65.71)	12 (34.29)		
Gender			0.2294	0.632
Male	47 (83.93)	9 (16.07)		
Female	28 (80.00)	7 (20.00)		
Level of hearing loss			0.9182	0.632
Moderate (41‑60)	2 (100)	0 (0)		
Severe (61‑80)	21 (77.78)	6 (22.22)		
Profound (>81)	52 (83.87)	10 (16.13)		

Table 5 showing the logistic regression analysis demonstrated a significant association between the level of hearing loss and dental caries presence (p = 0.027), suggesting that individuals with higher levels of hearing loss may be more prone to dental caries. However, age (p = 0.882) and gender (p = 0.859) did not exhibit significant associations with dental caries presence.

**Table 5:** Logistic Regression Analysis of Dental Caries Presence

	Coefficient	Std. Err.	z	P>|z|	95% Conf. Interval
Age group	0.070	0.468	0.15	0.882	(-0.848, 0.987)
Gender	-0.103	0.582	-0.18	0.859	(-1.245, 1.038)
Level of hearing loss	1.739	0.785	2.21	0.027	(0.200, 3.279)
Constant (_cons)	-6.051	2.975	-2.03	0.042	(-11.882, -0.221)

Table 6 shows that age group has a significant association with the presence of gingival bleeding (p = 0.001), while gender shows a trend towards significance (p = 0.090). However, the level of hearing loss does not appear to be significantly associated with gingival bleeding (p = 0.556). The overall model was statistically significant (p = 0.002), indicating that the predictors collectively explain a significant proportion of the variance in periodontal status.

**Table 6:** Logistic Regression Analysis of Gingival Bleeding Presence

	Coefficient	Std. Err.	z	P>|z|	95% Conf. Interval
Age group	2.246	0.691	3.25	0.001	(0.891, 3.601)
Gender	1.160	0.683	1.70	0.090	(-0.179, 2.500)
Level of hearing loss	0.358	0.609	0.59	0.556	(-0.835, 1.552)
Constant (_cons)	-9.743	3.236	-3.01	0.003	(-16.085, -3.401)

## Discussion

Everyone in the nation, including those with disabilities, has the same rights to maintain good oral health. In light of this, the current study was carried out to evaluate the oral health status among hard-of-hearing children in the Cuttack district of Odisha.

In our study, we found the prevalence of profound hearing loss (>81dB) to be higher among the group of the participated children which is found similar to a study done by Kalavani et al. [10]. The number of males in our study was 61%, higher than the number of females which explains the fact that some rural parts of India still believe that female child education does not help the family's economic prosperity. This is in contrast to a study done in Ethopia by Tefera et al. [11] in which majority of participants were female.

Maintaining appropriate oral hygiene practices is essential for optimal oral health, and it is best to start practicing these habits as early as childhood. Among the study participants by level of hearing loss, most of the children (96%) in the profound category brushed their teeth once daily followed by severe and moderate category.

In the present study, gingival bleeding was seen more (82%) among the patients. These results were consistent with another study done on children with disabilities in Nalgonda, India, where 91.7% of hearing-impaired students and 96% of the visually impaired children had calculus [12]. Consequently, the current study's conclusion that hard-of-hearing children had a higher prevalence of poor periodontal health (83%), may have resulted from the children's ongoing struggle to maintain good oral hygiene.

The responses they had for the frequency of toothache or feeling discomfort due to your teeth during the past 12 months also varied. Most of the children (67.03%) never had toothache and 22% of children had toothache occasionally in the last one year. Only 6.59% of children had toothache rarely. Most of the children (90%) never received or visited the dentist in the past one year. Most of the children (98%) were satisfied with the appearance of their teeth. Most of the children didn't have any difficulty in biting hard food while a few (eight) children had difficulty in chewing food.

In 8% of the study participants, dental fluorosis was observed. This result is in accordance with a study on hard-of-hearing and speech-impaired children that was carried out in Kuwait, where 10% of the participants had it [13]. The possible reason for fluorosis in our study was the chronic ingestion of excessive amounts of fluoride through fluoridated water, fluoride in food, dentifrices, etc.

The current study's analysis of the Decayed, Missing, and Filled Teeth (DMFT) yielded a mean value of 3.47 (±0.30), which is lower than studies conducted in Chennai (1.1) [14], Mysore (1.2) [15], Bhopal city (2.1) [16], Bangalore (2.1) [17] and Davangere (1.64) [18]. The mean in our study (3.47±3) was higher than that observed in the Udaipur study (2.61) [19].

Treatment needs of this differently abled study group showed 48% of the population needs prompt treatment including oral prophylaxis whereas 26% of the study population needs immediate or have urgent treatment needs due to trauma or infection of dental origin. These results are in accordance with the study done by Jain et al. (2008) [19], Rao et al. (2001) [20], and Hennequin et al. (2000) [21]. The factors influencing these accumulated treatment needs are lack of knowledge about good oral hygiene practices, lack of motivation, and low priority given to dental care in the society.

Another revelation was shown in the study stating that 4% of the study population were weekly tobacco chewers which may be due to lack of family ecosystem of the differently abled individuals or under surveillance of caretakers present in institutions where these children are educated.

The study reveals that hearing and speech impairment affect the oral health status of these children. Hence children with disabilities need more attention towards oral health care needs. There are a few drawbacks that need to be addressed, one is the small sample size, and a larger population can be taken in future studies. Also children's socio-economic background has not been considered as most of them were institutionalized. The questionnaires were answered by the caregiver and hence there is a potential risk for response bias. Also there were other confounding variables such as sociodemographic variables of parents which could not be taken as it was institution based and hence it needs to be considered for future research.

These results highlight the need for comprehensive and customized oral health treatments created to address the unique requirements of this population and ultimately improve their quality of life.

## Conclusions

In conclusion, this study among children with disabilities shows a high prevalence of dental caries, periodontal problems and unmet dental treatment needs with low attendance to dental services. Improving oral health can start with promoting effective tooth brushing from an early age, and schools are ideal environments for teaching oral health practices. Classroom-based oral health education led by teachers can play a significant role in enhancing oral care. School dental health initiatives should emphasize preventive strategies like fluoride mouth rinses and regular tooth brushing, beginning early to safeguard primary teeth and prevent cavities in permanent ones.

Since children with hearing and speech impairment have specific needs, further research is essential to develop comprehensive oral health programs and ensure the effective implementation of the National Oral Health Program that includes children with special health care needs (CSHCN). The development of Information, Education, and Communication materials, particularly those featuring visual aids and Indian Sign Language, could greatly support oral health promotion among hard-of-hearing children.

## Figures and Tables

**Figure 1:**
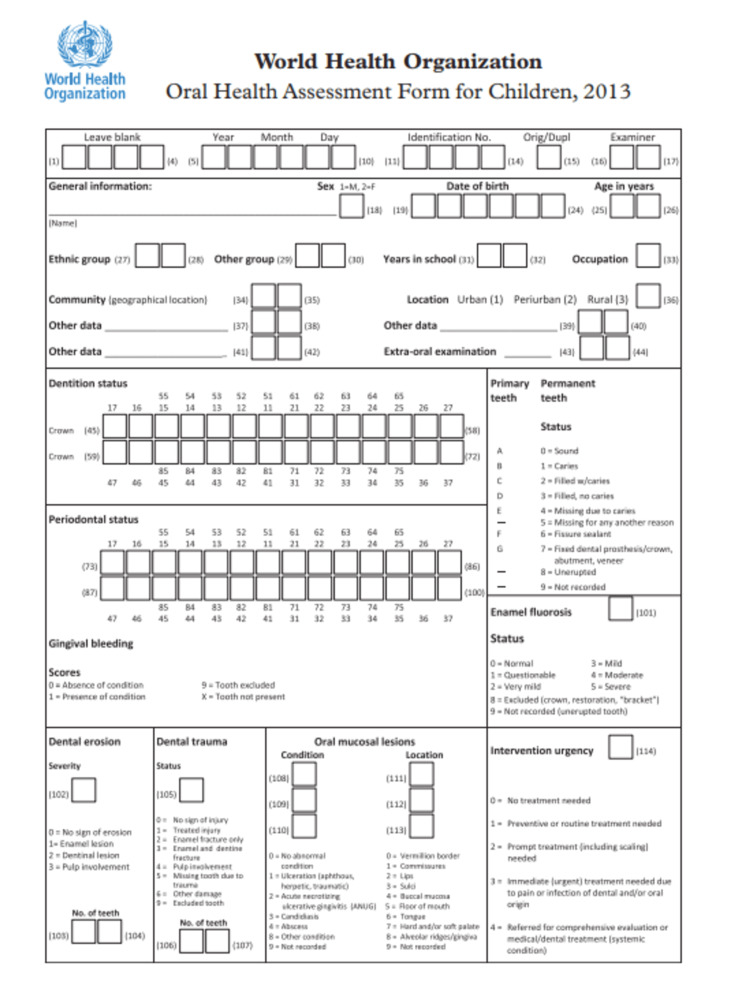
WHO Oral health assessment form for children 2013

**Figure 2:**
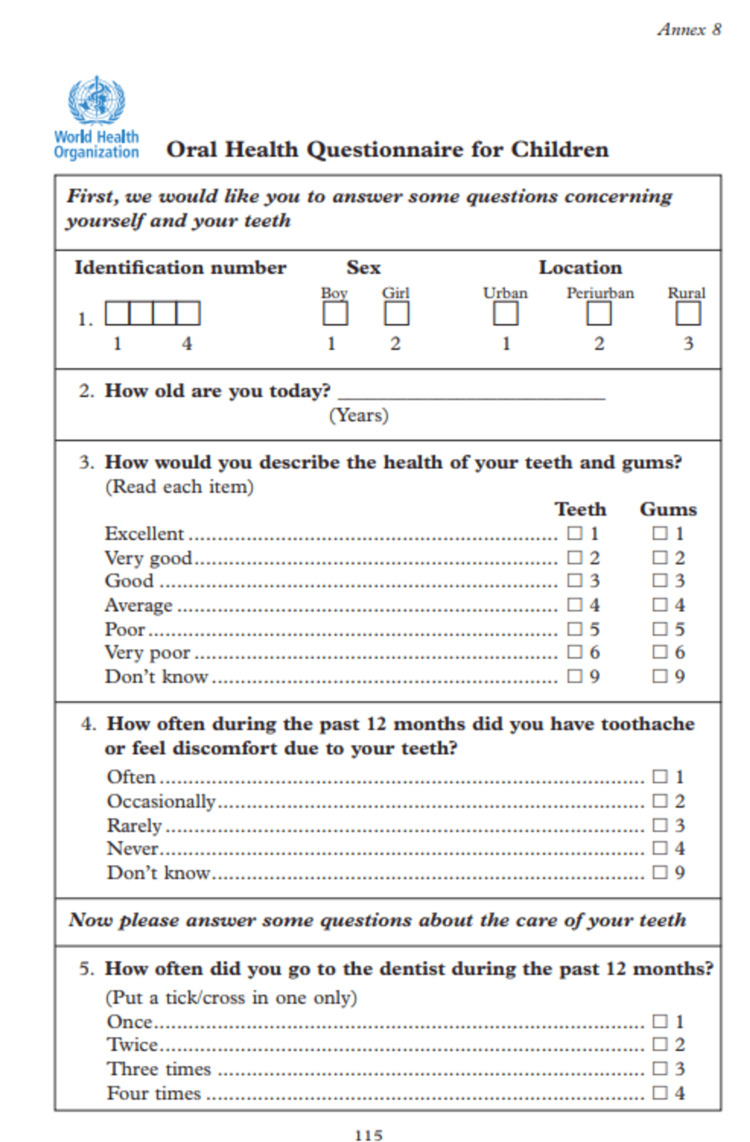
WHO Oral health questionnaire for children (1)

**Figure 3:**
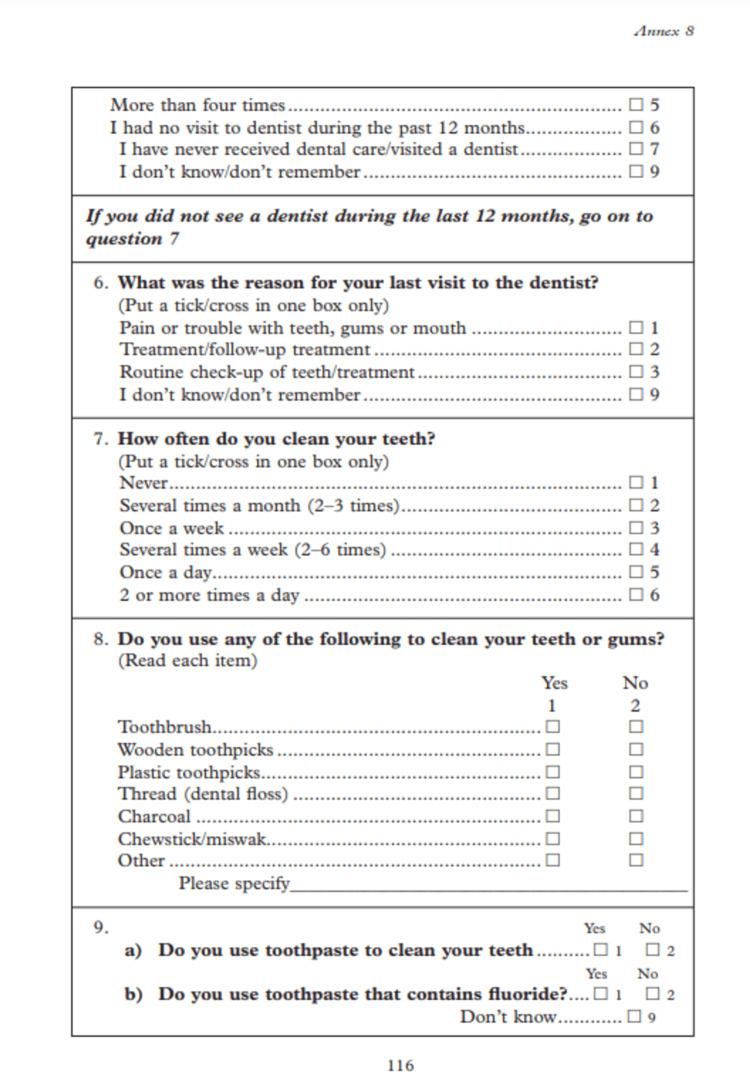
WHO Oral health questionnaire for children (2)

**Figure 4:**
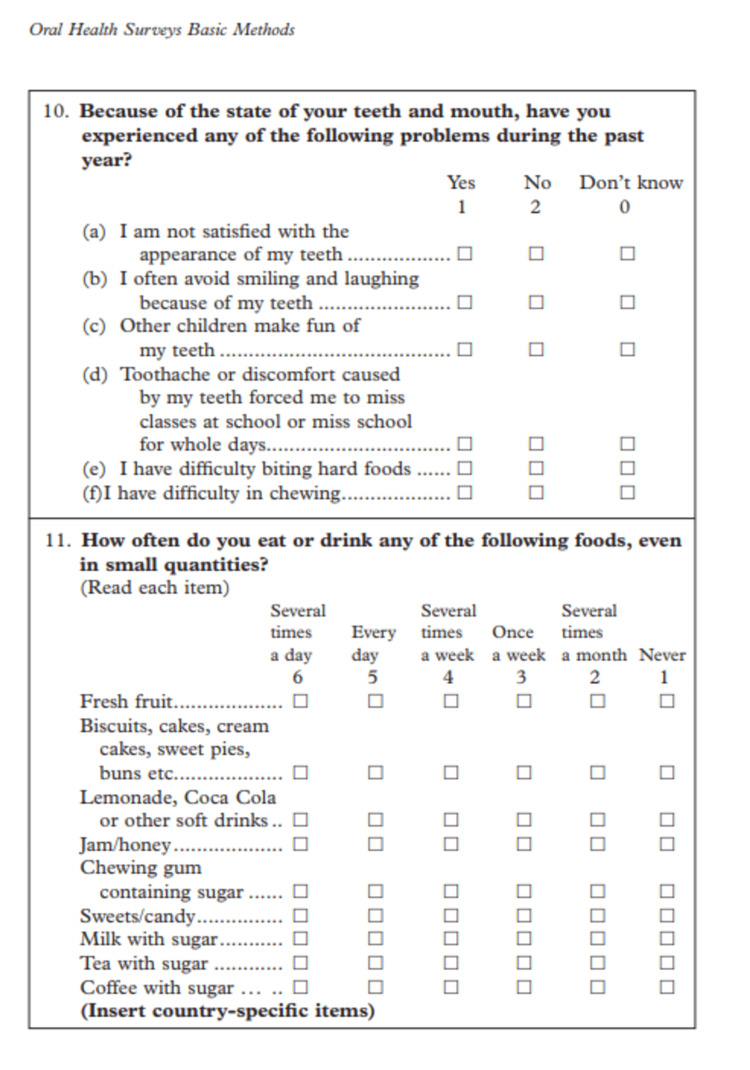
WHO Oral health questionnaire for children (3)

## Appendices

Appendix 1

Appendix 2

Appendix 3

Appendix 4
